# A129 OUTCOMES FOLLOWING ENDOSCOPIC SUBMUCOSAL DISSECTION OF SUPERFICIAL GASTROINTESTINAL TRACT NEOPLASTIC LESIONS FROM A TERTIARY-CARE ACADEMIC CENTRE

**DOI:** 10.1093/jcag/gwad061.129

**Published:** 2024-02-14

**Authors:** S Gupta, F Dang, Y Fujiyoshi, S Li, K Khalaf, G May, J Mosko, C Teshima

**Affiliations:** Medicine, University of Toronto, Toronto, ON, Canada; Medicine, University of Toronto, Toronto, ON, Canada; Division of Gastroenterology, University of Toronto, Toronto, ON, Canada; University of Calgary, Calgary, AB, Canada; Division of Gastroenterology, St Michael's Hospital, Toronto, ON, Canada; Division of Gastroenterology, St Michael's Hospital, Toronto, ON, Canada; Division of Gastroenterology, St Michael's Hospital, Toronto, ON, Canada; Division of Gastroenterology, St Michael's Hospital, Toronto, ON, Canada

## Abstract

**Background:**

Endoscopic submucosal dissection (ESD) is an advanced luminal resection technique for dysplastic and early cancerous gastrointestinal (GI) tract neoplasms. By removing lesions en bloc, this method provides more accurate histopathologic evaluation of resection margins in comparison to endoscopic mucosal resection (EMR). Superior curative resection and lower recurrence rates over EMR have been shown in addition to the benefit of reduced morbidity and mortality compared to surgery. While ESD has become well-established in Asia and Europe, its adoption in North America – and in particular Canada – has been slowed by multiple technical and economic factors including a scarcity of infrastructure, institutional support, and standardized training. Additionally, reproduced Canadian data corroborating its known global efficacy is currently lacking.

**Aims:**

Our aim was to evaluate clinical and technical outcomes in patients who underwent ESD of superficial GI tract neoplasms at a tertiary-care academic centre.

**Methods:**

We conducted a retrospective single-centre cohort study at The Centre for Advanced Therapeutic Endoscopy & Endoscopic Oncology, St. Michael’s Hospital, Toronto, Canada. Consecutive adults (age ampersand:003E 18) who underwent ESD of any superficial GI tract neoplasm from October 2017 to December 2022 were included in the study. Primary outcomes were rates of en bloc, complete (R0), and curative resections. Secondary outcomes were rates of recurrence and adverse events. Data was collected through electronic chart review.

**Results:**

ESD was performed in a total of 308 lesions with a mean size of 2.5 cm (range, 1-7 cm). Gastric ESD was the most common (n=130), followed by esophageal (n=91), rectal (n=62), and colonic (n=22). Three patients underwent duodenal ESD. En bloc, R0, and curative resection rates were 89.2%, 84.7%, and 71.8%, respectively. 64.3% of lesions were cancerous on pathology (n=198), of which 48 had at least submucosal invasion. Rates of delayed bleeding and deep mural injury (DMI) requiring surgical intervention were 3.9% and 1.3%, respectively. Overall recurrence at the first surveillance endoscopy (SE1) was 3.6%.

**Conclusions:**

En bloc, R0, and curative resection rates reported from this largest single-centre Canadian ESD cohort are comparable to existing North American data. Low rates of early recurrence and an adequate safety profile have been demonstrated with this emerging oncologic method of resection. Additional investigation is warranted to optimize lesion selection and determine long-term clinical outcomes to further establish this technique in the management of superficial GI tract neoplasms.

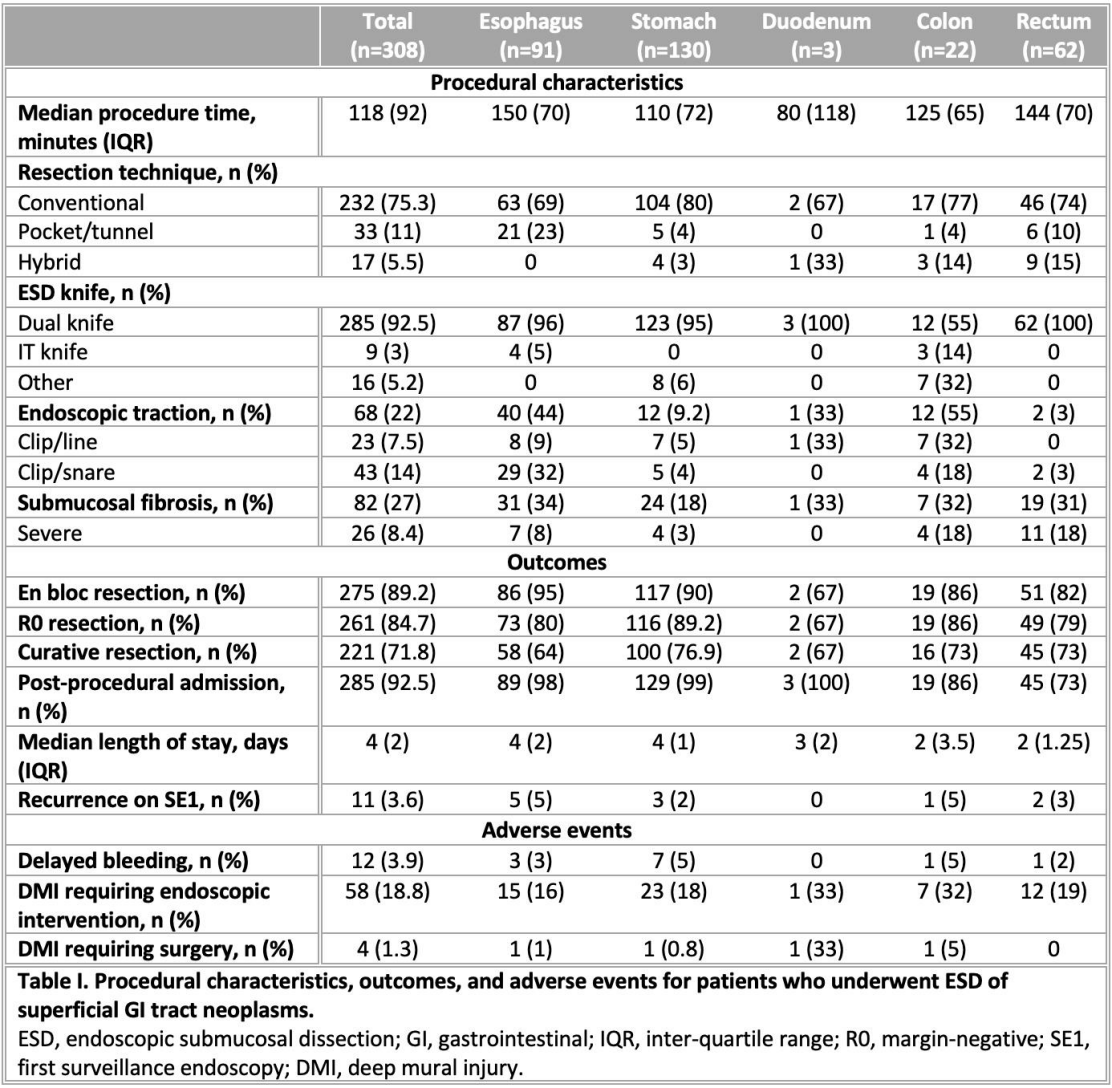

Table I. Procedural characteristics, outcomes, and adverse events for patients who underwent ESD of superficial GI tract neoplasms.

**Funding Agencies:**

None

